# Comprehensive Analysis of the Mechanism of Anoikis in Hepatocellular Carcinoma

**DOI:** 10.1155/2024/8217215

**Published:** 2024-09-11

**Authors:** Dongqian Li, Qian Bao, Shiqi Ren, Haoxiang Ding, Chengfeng Guo, Kai Gao, Jian Wan, Yao Wang, MingYan Zhu, Yicheng Xiong

**Affiliations:** ^1^ Department of Hepatobiliary and Pancreatic Surgery Affiliated Hospital of Nantong University Medical School of Nantong University, Nantong 226001, Jiangsu, China; ^2^ Nantong University Medical School, Nantong 226001, Jiangsu, China

## Abstract

**Background:**

Hepatocellular carcinoma (HCC), ranking as the second-leading cause of global mortality among malignancies, poses a substantial burden on public health worldwide. Anoikis, a type of programmed cell death, serves as a barrier against the dissemination of cancer cells to distant organs, thereby constraining the progression of cancer. Nevertheless, the mechanism of genes related to anoikis in HCC is yet to be elucidated.

**Methods:**

This paper's data (TCGA-HCC) were retrieved from the database of the Cancer Genome Atlas (TCGA). Differential gene expression with prognostic implications for anoikis was identified by performing both the univariate Cox and differential expression analyses. Through unsupervised cluster analysis, we clustered the samples according to these DEGs. By employing the least absolute shrinkage and selection operator Cox regression analysis (CRA), a clinical predictive gene signature was generated from the DEGs. The Cell-Type Identification by Estimating Relative Subsets of RNA Transcripts (CIBERSORT) algorithm was used to determine the proportions of immune cell types. The external validation data (GSE76427) were procured from Gene Expression Omnibus (GEO) to verify the performance of the clinical prognosis gene signature. Western blotting and immunohistochemistry (IHC) analysis confirmed the expression of risk genes.

**Results:**

In total, 23 prognostic DEGs were identified. Based on these 23 DEGs, the samples were categorized into four distinct subgroups (clusters 1, 2, 3, and 4). In addition, a clinical predictive gene signature was constructed utilizing ETV4, PBK, and SLC2A1. The gene signature efficiently distinguished individuals into two risk groups, specifically low and high, demonstrating markedly higher survival rates in the former group. Significant correlations were observed between the expression of these risk genes and a variety of immune cells. Moreover, the outcomes from the validation cohort analysis aligned consistently with those obtained from the training cohort analysis. The results of Western blotting and IHC showed that ETV4, PBK, and SLC2A1 were upregulated in HCC samples.

**Conclusion:**

The outcomes of this paper underscore the effectiveness of the clinical prognostic gene signature, established utilizing anoikis-related genes, in accurately stratifying patients. This signature holds promise in advancing the development of personalized therapy for HCC.

## 1. Introduction

In the context of liver cancer, the most common malignant tumor is hepatocellular carcinoma (HCC), which is its predominant subtype. It ranks second in global mortality rates and represents the fourth major cause of mortality worldwide [1, 2]. This underscores the substantial burden HCC places on global public health [3]. Recent advances in therapeutic strategies have contributed to a gradual reduction in both the incidence and mortality rates associated with HCC. However, addressing the high postoperative recurrence rate and the relatively low five-year survival rate of HCC remains imperative [4, 5]. HCC progression is characterized by distinct early and late stages. The accurate risk stratification of patients with HCC is crucial for determining the clinical prognosis and treatment recommendations for individuals with HCC. Despite the development of multiple staging and prognostic systems, achieving consensus on a unified survival-prediction system has proven elusive [6–8]. Therefore, elucidating the molecular mechanisms underlying HCC development, screening potential tumor-specific biomarkers, and establishing novel prognostic models are critical for risk stratification, early diagnosis, treatment, and personalized therapy development for patients with HCC.

Anoikis, a type of programmed cell death, is initiated by the disruption of the interaction between cells and extracellular matrix (ECM) [9, 10]. Prior research has reported that anoikis is critically involved in cancer cell invasion and metastasis [11, 12]. Anoikis can prevent cancer cell dissemination to distant organs, limiting cancer progression [13]. In the course of tumor progression, a protective barrier shields tumor cells, exerting protective effects against cell death by inhibiting the activation of molecules that initiate anoikis. This confers resistance against anoikis and promotes tumor cell survival [14, 15]. Therefore, multiple types of invasive metastatic cancer may acquire and maintain anoikis resistance through different mechanisms. Previous studies have reported that the activated NF-*κ*B regulates the PI3K/Akt pathway, a survival-promoting pathway that significantly contributes to tumor growth and metastasis. Thus, targeting the PI3K/AKT pathway emerges as a viable therapeutic strategy for cancer [16, 17]. In addition, the PI3K/AKT pathway can suppress anoikis through the inhibition of BCL2L11, BAD, and TMPRSS9 by promoting their phophorylation [12, 16]. NTRK2, a type of NF-*κ*B, is an antiapoptotic molecule that confers anoikis resistance. Previous research has reported the involvement of NTRK2 in the development and metastasis of multiple cancer types, encompassing cervical, colorectal, and gastric cancers [18–20]. Furthermore, Mak et al. suggested that miR-141 can upregulate surviving in ovarian cancer by downregulating KLF12, contributing to tumor metastasis and anoikis resistance [21]. Nevertheless, the underlying mechanisms and pathways regulating anoikis in the advancement and invasion of HCC remain unclear. It is imperative to unravel the mechanisms conferring resistance to anoikis in HCC to enhance treatment outcomes and prognosis for patients with this disease.

This investigation employed bioinformatics analysis to identify pivotal regulatory factors associated with anoikis in HCC. Anoikis-associated genes linked to the prognosis were clustered, and different anoikis-related subgroups were identified using unsupervised clustering algorithms [22]. Different subgroups underwent Kyoto Encyclopedia of Genes and Genomes (KEGG) pathway enrichment analysis to elucidate potential biological pathways associated with their distinct characteristics. Finally, prognostic genes were screened, and a prognostic risk model was constructed utilizing the least absolute shrinkage and selection operator (LASSO) regression, as well as both univariate and multivariate Cox regression analyses. This research highlighted that the predictive model, utilizing genes related to anoikis, could stratify the risk among patients with HCC, accurately predicting their prognosis. This model contributes significantly to the clinical management and personalized treatment strategies for individuals with HCC.

## 2. Methods

### 2.1. Acquisition and Processing of Data

The integration of comprehensive data resources was fundamental to this research, wherein RNA sequencing (RNA-seq) data, along with clinical details of individuals diagnosed with HCC, were retrieved from the Cancer Genome Atlas (TCGA). TCGA-HCC cohort comprised the data of 50 noncancerous samples and 374 HCC samples. Moreover, the GSE76427 dataset, comprising 115 HCC and 52 noncancerous samples, was acquired from the Gene Expression Omnibus (GEO). TCGA-HCC and GEO datasets were merged to obtain the HCC dataset. The batch effects in various datasets were eliminated using the ComBat function with the R “SVA.” The HCC cohort was randomly split into validation and training groups by the “caret.” The TCGA database provided data on somatic mutation and copy number variation (CNV) for individuals with HCC. Genes related to anoikis were sourced from GeneCards [23].

### 2.2. Unsupervised Clustering Analysis

The isolation of expression levels for genes related to anoikis within the TCGA-HCC cohort preceded the determination of differentially expressed genes (DEGs) linked to anoikis in the comparative analysis between HCC and noncancerous samples. Subsequently, unsupervised clustering analysis, facilitated by the R “ConsensusClusterPlus,” was utilized to unveil expression patterns of these DEGs within the HCC cohort. Differential expression analysis between distinct subtypes was then conducted utilizing the R “limma.”

### 2.3. Functional Enrichment Analysis and Gene Set Variation Analysis (GSVA)

The gene sets labeled as “c2. cp. kegg. v6.2. symbols” were retrieved from MSigDB and used in GSVA via the R “GSVA” to discern the differential biological pathways between different subgroups of anoikis [24]. The R “clusterProfiler” was utilized with the significance criterion *P* < 0.05 to screen for potential biological processes and KEGG pathways enriched with the DEGs [25]. The Gene Set Enrichment Analysis (GSEA) software was utilized to conduct a differential KEGG pathway analysis, comparing the pathways between high-risk (HR) and low-risk (LR) groups. The nominal *P* value and normalized enrichment score (NES) were determined to analyze enrichment levels and significance, respectively.

### 2.4. Single-Sample GSEA (ssGSEA)

The ssGSEA algorithm was applied to determine the infiltration level of 23 immune cells in HCC and their correlation with the NES.

### 2.5. Establishment and Verification of a Prognostic Gene Signature

DEGs underwent univariate CRA to determine anoikis-associated genes with prognostic value as per the criterion of *P* < 0.05. Through the LASSO Cox regression model, the candidate genes were further narrowed down using the LASSO Cox regression model to develop prognostic gene signatures. The risk score was derived as mentioned in the following.

Risk score = (gene 1 expression level × *β*1) + (gene 2 expression level × *β*2) + (gene 3 expression level × *β*3) + … + (gene *N* expression level × *βN*), where *β* depicts the regression coefficient.

The training and validation sets have a median risk score. In this case, the patients diagnosed with HCC were classified into risk groups, specifically LR and HR.

A comparison of the survival rates of individuals in both risk groups was carried out utilizing Kaplan–Meier (KM) curve analysis. The visualization of risk gene expression levels in these groups was accomplished through the application of heatmaps. The model's predictive performance was assessed through the (a) ROC (receiver operating characteristic) curve analysis and (b) the AUC (area under the ROC curve). By performing multivariate and univariate CRA, we established the risk score's prognostic significance, which is independent.

### 2.6. Nomogram Development

We devised a nomogram model through the amalgamation of the risk score alongside various clinical characteristics. Moreover, in view of the HCC patients, we used the nomogram in conjunction with the “rms” and “survival” tools to forecast their 5-, 3-, and 1-year survival rates. The utilization of the calibration curve allowed for the assessment of the precision and distinguishing ability of the nomogram.

### 2.7. Tumor Microenvironment (TME) Infiltration and Somatic Mutations in Distinct Risk Groups

Concerning the RNA (CIBERSORT) algorithm, the approximation of its relative subsets took place. Alternatively, it is also called cell-type identification. Accordingly, we identified the risk score's link to infiltrating immune cells. The R “maftools” was utilized to assess somatic cell variation. The waterfall plot was employed to present the mutation profile of individuals with HCC in both risk groups.

### 2.8. Drug Sensitivity Analysis

The drug response was predicted using the Cancer Drug Sensitivity Genomics database with the R “pRRophic”. Ridge regression was employed for estimating the half-maximal inhibitory concentration (IC50) values of drugs for all individuals. Estimation of the predictive accuracy was achieved through 10-fold cross-validation [26].

### 2.9. Single-Cell RNA-Seq Data Analysis

The single-cell RNA-seq dataset GSE166635, encompassing the data of two HCC samples, was retrieved from GEO. The R “Seurat” was used to preprocess the original dataset, ensuring data quality. Subsequently, cells were clustered utilizing filtered principal components. For visual classification purposes, the dimensionality reduction techniques of the Unified Manifold Approximation and Projection (UMAP) were used. With a threshold of corrected *P* < 0.05, with regard to the immune cells, the screening of marker genes was carried out. Following that, to ascertain the category group of immune cells, we extracted marker genes associated with immune cells from PanglaoDB and cross-referenced them with the genes of every category group. Finally, this paper examined the risk genes' correlation with the single-cell subpopulation.

### 2.10. Western Blotting

Cells in the culture flask were subjected to a phosphate-buffered saline (PBS) rinse, followed by lysis using ice-cold lysis buffer (Solarbio, China). The resulting lysates underwent centrifugation at 12,500 rpm and 4°C. The supernatant was boiled in the supersampling buffer for 10 minutes. Following that, the protein samples underwent the process of sodium dodecyl sulfate-polyacrylamide gel electrophoresis, utilizing a gel with a concentration of 12.5%. Subsequently, the separated proteins were transferred onto a polyvinylidene difluoride membrane, characterized by a specific pore size of 0.2 *µ*m. To block nonspecific binding sites, the membrane was exposed to 5% skim milk. Following the aforementioned steps, the membrane underwent an overnight incubation on a shaker at 4°C. During this incubation, primary antibodies, including anti-ETV4, anti-PBK, anti-SLC2A1 (all 1 : 1000, CST), and antiaction (1 : 5000, CST) were applied. The appropriate dilutions were made following the provided guidelines to ensure accurate and effective antibody-antigen interactions. Subsequently, the membrane underwent a subsequent phase involving a 2-hour incubation at room temperature. During this interval, secondary antibodies were employed, and the incubation was carried out with a slow shaking technique. After washing the membrane thrice with PBS, immunoreactive signals were developed using the ECL Plus kit. Normalization of the expression levels of target proteins to the corresponding levels of action was conducted.

### 2.11. Clinical Specimen Collection and Immunohistochemical (IHC) Analysis

This study obtained eight paired HCC tumor tissues and their corresponding noncancerous lung tissues from the Affiliated Hospital of Nantong University. Approval of this investigation was provided by the Ethics Committee of Nantong University Hospital. The provision of informed consent was considered necessary for inclusion in the study and for the use of the samples.

The sections were dewaxed and subjected to antigen retrieval. Following blocking with bovine serum albumin, the sections were exposed to anti-PDXK primary antibodies (1 : 200) at 4°C overnight, followed by three rinses with PBS. Subsequently, the sections underwent incubation with secondary antibodies for 30 minutes at 37°C. Then, the sections underwent staining with 3,3′-diaminobenzidine for 5–10 minutes and hematoxylin for 10 seconds. IHC staining scores were calculated as per the intensity and amount of staining. The staining intensity was assessed utilizing the following distinct scores: score 0 signifying a negative result, score 1 signifying a weak intensity, score 2 signifying a moderate intensity, and score 3 representing a strong intensity. In addition, the scoring system for immunopositive cell proportions ranged from 1 to 4, with score 1 signifying 0–25% immunopositive cells, score 2 signifying 26–50% immunopositive cells, score 3 signifying 51–75% immunopositive cells, and score 4 signifying 76–100% immunopositive cells. This comprehensive scoring methodology aimed to capture both the intensity and extent of immunopositivity, providing a nuanced assessment of the staining results.

### 2.12. Statistical Analysis

R v 4.01 was used for all statistical analyses. The comparison of means between two groups was conducted through the Wilcoxon test, while the comparison of means among multiple groups was performed utilizing the Kruskal–Wallis test. In addition, the survival rates among various groups were compared using KM analysis and the logarithmic rank test. Pearson correlation analysis was utilized to verify the correlation. The “limma” package was utilized to construct principal component analysis (PCA) plots for different subgroups and risk groups. For ROC curve analysis, the R “survival” and “timeROC” were utilized. The statistical significance was set at *P* < 0.05.

## 3. Results

### 3.1. Anoikis-Related Gene Expression and Variation in HCC

**Figure 1:**
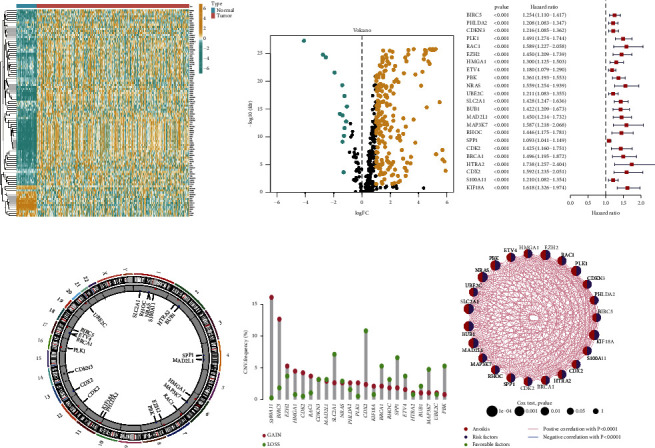
Expression and genetic changes of anoikis-related genes in hepatocellular carcinoma (HCC). (a-b) The differentially expressed genes (DEGs) between HCC and noncancerous samples in the cancer genome atlas (TCGA)-HCC cohort were visualized using heatmap and volcanic maps. (c) Anoikis-related prognostic genes in the HCC cohort. (d) The changes in the copy number variation of anoikis-related regulatory factors on chromosomes. (e) CNV frequency of anoikis-related regulatory factors. The height of the column represents the frequency of change. Missing frequency, green dot; amplification frequency, red dot. (f) This network shows the interactions between anoikis-related genes.

From the TCGA-HCC dataset, 217 anoikis-related DEGs were extracted (Figures 1(a) and 1(b)). In the HCC cohort, 208 anoikis-related genes were acquired utilizing 217 DEGs. Univariate Cox regression analysis revealed 23 anoikis-related prognostic genes (Figure 1(c)). Figure 1(d) illustrates the changes in the CNV of anoikis-related regulatory factors. Among the 23 anoikis-related regulatory factors, genes such as S100A11, BIRC5, EZH2, HMGA1, CDK2, and RAC1 exhibited significant copy number amplification, whereas genes such as SLC2A1, CDX2, BRCA1, RHOC, SPP1, ETV4, MAP3K7, and PBK exhibited significant copy number deletion (Figure 1(e)). Figure 1(f) illustrates the interaction correlation between 23 anoikis-related genes.

### 3.2. Identification of Anoikis-Related Subgroups in HCC

**Figure 2:**
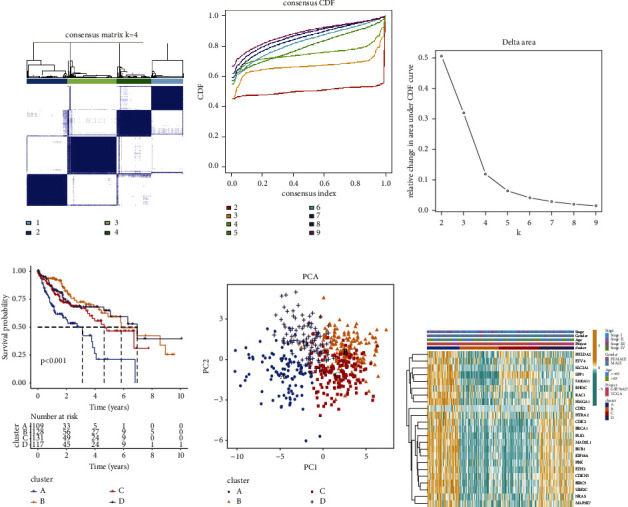
Identification of anoikis-related subgroups in hepatocellular carcinoma (HCC). (a–c) The consistency clustering algorithm was used to classify HCC samples into four different subgroups (cluster A, cluster B, cluster C, and cluster D). C. Consistency matrix; D. Cumulative distributive function (CDF) diagram; E. *K* = 2–9. The relative change in area under the CDF curve at time F. (d) The overall survival rate in the four subgroups. (e) Principal component analysis of four subgroups. (f) The expression levels of anoikis-related genes in four subgroups.

Unsupervised cluster analysis based on 23 anoikis-related genes revealed four subgroups (clusters A, B, C, and D) (Figures 2(a), 2(b), and 2(c)). The KM survival curve shows the survival rates of patients in the four subgroups. Cluster A was associated with the worst survival outcome (Figure 2(d)). PCA demonstrated that the four subgroups exhibited distinct clustering (Figure 2(e)). The heatmap revealed the differential gene expression levels among clusters A, B, C, and D (Figure 2(f)).

### 3.3. Correlation between Different Subgroups and Immune Cell Infiltration and the Biological Pathways between Distinct Subgroups

**Figure 3:**
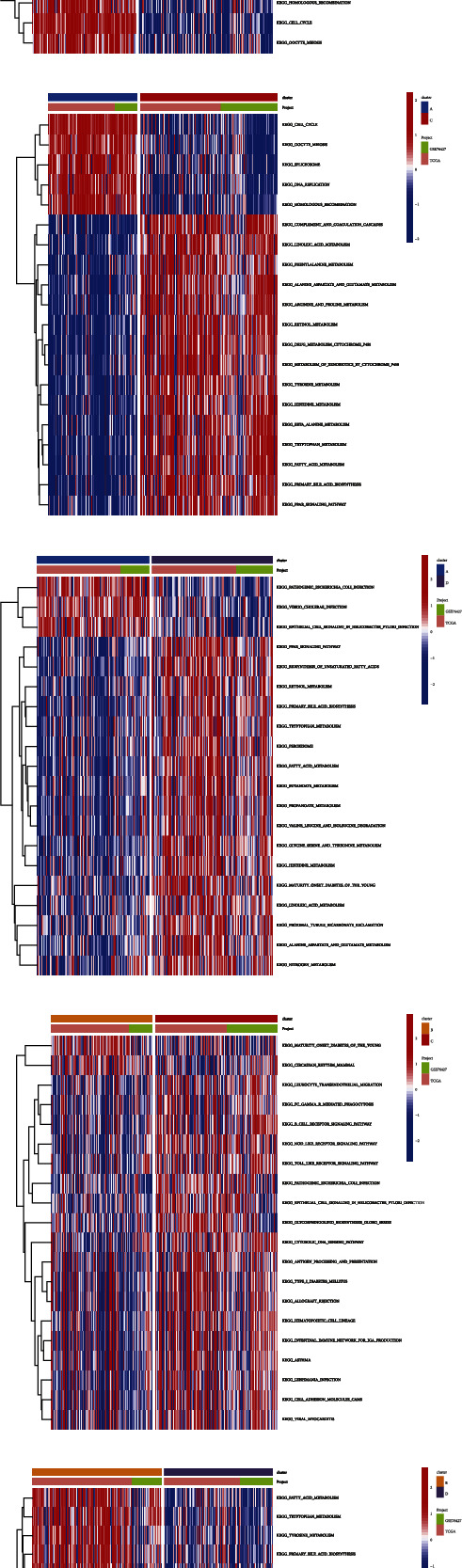
The differential immune landscape characteristics and biological pathways between hepatocellular carcinoma (HCC) subtypes. (a) The differential abundances of infiltrating immune cells in the tumor microenvironment between HCC subgroups. (b–g) Gene set variation analysis revealed the activation status of biological pathways in different subgroups. Heatmaps are used to visualize these biological processes (red and blue represent activated and inhibited pathways, respectively). B: cluster A vs. cluster B; C: cluster A vs. cluster C; D: cluster A vs. cluster D. E: cluster B vs. cluster C; F: cluster B vs. cluster D; G: cluster C vs. cluster D.

The tumor-infiltrating immune cells in the four subgroups were analyzed. The immune cell infiltration levels significantly varied between the four subgroups (Figure 3(a)). GSVA was executed to examine the differential biological pathways among the four subgroups. Cluster A was remarkably correlated with various pathways, including PPAR signaling and peroxisome pathways. Cluster B was significantly associated with pathways, including homologous recombination and cell cycle. Cluster C was significantly correlated with metabolic pathways, including tyrosine, histidine, and tryptophan metabolism. Cluster D was significantly associated with energy metabolism pathways, including fatty acid metabolism and nitrogen metabolism (Figures 3(b), 3(c), 3(d), 3(e), 3(f), and 3(g)).

### 3.4. Construction of Prognostic Gene Signature

**Figure 4:**
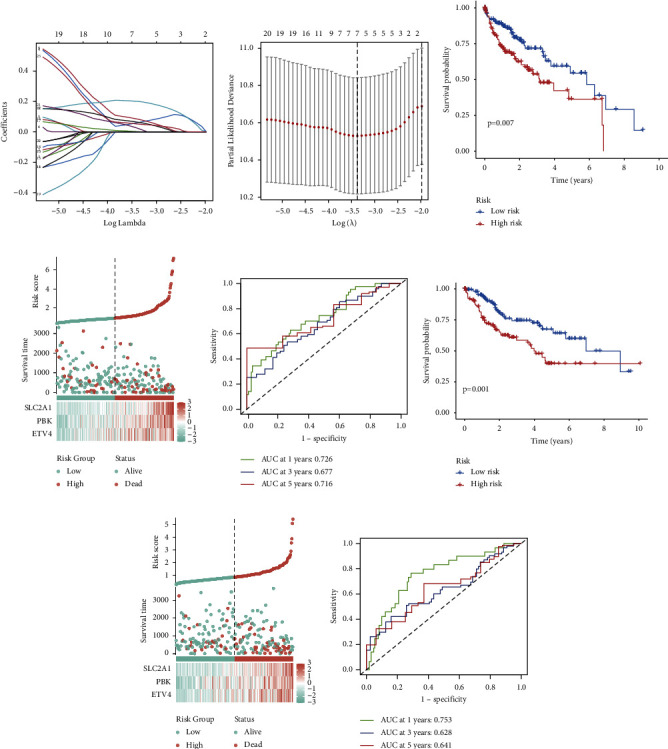
Construction of a clinical prognosis model. (a) The coefficient distribution of anoikis-related prognostic genes from least absolute shrinkage and selector operator (LASSO) regression analysis. (b) Ten-fold cross-validation of tuning parameter selection in LASSO analysis. (c) Differential survival rates between the high-risk and low-risk groups. (d) The distribution of risk scores and survival status among different risk groups in the training group, as well as the expression levels of risk genes in the high-risk and low-risk groups. (e) Receiver operating characteristic (ROC) curve analysis for predicting the 1-year, 3-year, and 5-year survival rates in the training group. (f) Differential survival rates between the high-risk and low-risk groups in the validation group. (g) The distribution of risk scores and survival status among different risk groups in the validation group, as well as the expression levels of risk genes in the high-risk and low-risk groups. (h) ROC curve analysis for predicting the 1-year, 3-year, and 5-year survival rates in the training group.

A clinical prognostic gene signature was constructed as per 23 anoikis-related prognostic genes. Finally, three genes (ETV4, PBK, and SLC2A1) were identified to generate the gene signature (Figures 4(a) and 4(b)). The risk score was derived as follows. In this case, “expression level” is written as EL: risk score = ((*ETV4* EL × 0.1303) + (*PBK* EL × 0.2025) + (*SLC2A1* EL × 0.2592)).

Subjects were grouped according to their risk, specifically low or high. As observed in the training cohort, this approach follows the median risk score. KM analysis revealed that the overall survival (OS) of LR individuals was elevated relative to the HR individuals (*P*=0.007) (Figure 4(c)). Scores of individuals under the latter displayed a reduced probability of survival and poorer survival status relative to those under the former. The heatmap illustrated remarkable variations in the expression of risk genes between two risk groups (Figure 4(d)). The corresponding AUC values predicting 5-, 3-, and 1-year OS rates across were 0.716, 0.677, and 0.726, respectively (Figure 4(e)).

Based on the risk scores within the validation cohort, individuals were sorted according to their risks, specifically HR or LR. Those in the former exhibited lower OS rates in comparison to individuals in the latter (*P*=0.001) (Figure 4(f)). Individuals with HR scores exhibited a lower probability of survival and a worse survival status relative to those exhibiting LR scores. The heatmap indicated notable variations in risk gene expression between the two risk groups (Figure 4(g)). The corresponding AUC values to predict the 5-, 3-, and 1-year OS were 0.641, 0.628, and 0.753, respectively (Figure 4(h)).

### 3.5. Establishment of the Nomogram

**Figure 5:**
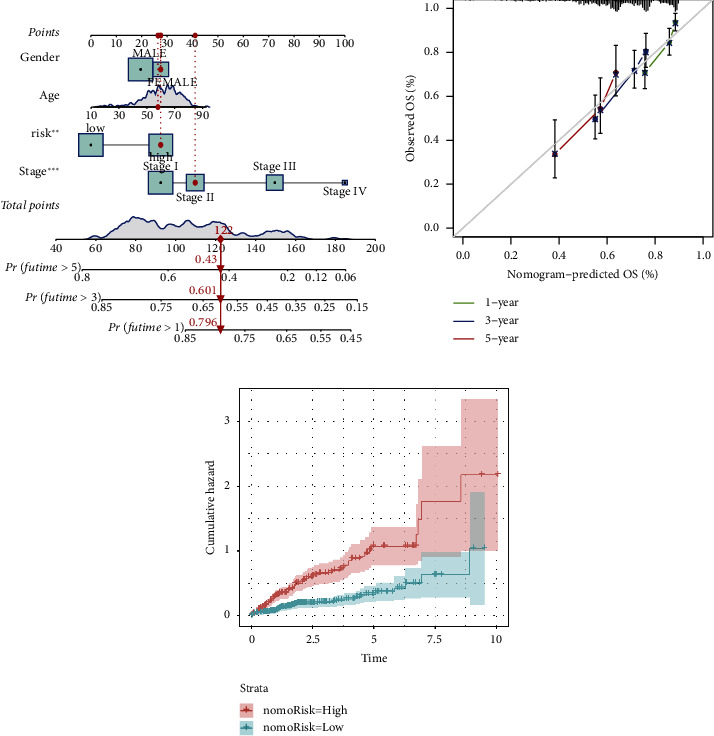
Construction and evaluation of a prognostic nomogram for hepatocellular carcinoma (HCC). (a) A nomogram was established to evaluate the 1-year, 3-year, and 5-year survival rates. (b) The calibration curve of the nomogram. (c) Cumulative incidence rate based on risk stratification.

A nomogram, combining risk scores and other clinical pathological parameters, was constructed to predict the 5-, 3-, and 1-year OS of individuals with HCC, aiming to enhance the predictive accuracy of the gene signature (Figure 5(a)). Calibration curves for 5-, 3-, and 1-year survival were constructed (Figure 5(b)). The cumulative risk for HR individuals was substantially higher than those with LR (Figure 5(c)).

### 3.6. Association between Gene Signature and Immune Cell and Somatic Cell Mutations

**Figure 6:**
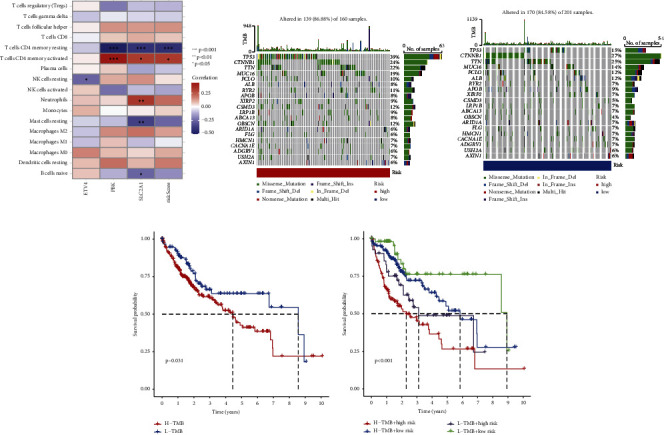
The correlation between prognostic gene signature and immune cell and somatic cell mutations. (a) The correlation between the risk genes and immune cells (b-c) was represented as a waterfall plot of tumor somatic mutations between the high-risk and low-risk groups. Each upper bar chart displays tumor mutation burden (TMB), while the number on the right represents the mutation frequency of each gene. The columns represent individual patients. B: high-risk group; C: low-risk group. (d) The differential overall survival rates between the high-TMB and low-TMB groups. (e) The overall survival of the low-TMB group was higher than that of the high-TMB.

Subsequently, we delved into the correlation of risk genes with immune cells. The ETV4 expression showed a negative link to the proportion of resting natural killer cells. PBK expression displayed a considerable negative association with the proportions of immune cells, encompassing resting memory CD4+ T cells. Meanwhile, PBK expression exhibited a remarkable positive association with the proportions of immune cells, such as resting memory CD4+ T cells. SLC2A1 expression exhibited a notable negative correlation with the proportions of immune cells, such as resting memory CD4+ T cells and resting mast cells. In contrast, SLC2A1 expression displayed a considerable positive association with the percentage of different immune cells, encompassing neutrophils and activated memory CD4+ T cells (Figure 6(a)). The differential somatic mutation profiles between the LR and HR groups in the TCGA-HCC cohort were examined utilizing the “maftools.” As depicted in the figure, the tumor mutation burden (TMB) in HR individuals surpassed that in low-risk individuals. Furthermore, TP53 mutation frequency was 39% in the HR individuals and 15% in the LR individuals (Figures 6(b) and 6(c)). The survival outcomes were more favorable for individuals with low TMB in comparison to those with high TMB (*P*=0.031) (Figure 6(d)). In addition, the OS of individuals with low TMB was elevated relative to the individuals with high TMB (*P* < 0.001) irrespective of HR or LR groups (Figure 6(e)).

### 3.7. Drug Sensitivity Analysis

**Figure 7:**
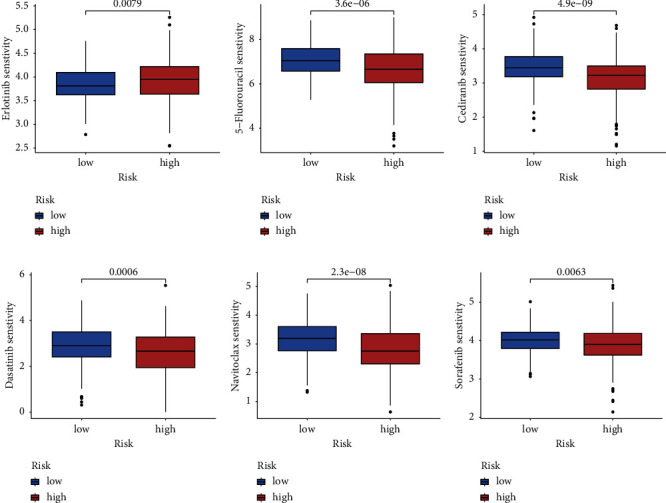
Drug sensitivity analysis. (a) The half-maximal inhibitory concentration (IC50) values of erlotinib, 5-fluorouracil, cediranib, dasatinib, navitoclax, and sorafenib varied between the low-risk and high-risk groups. (a) Erlotinib; (b) 5-fluorouracil; (c) cediranib; (d) dasatinib; (e) navitoclax; (f) sorafenib.

To evaluate the therapeutic effects of chemotherapy drugs and targeted drugs in individuals with HCC in both risk groups, the IC50 values of various commonly used drugs were quantified using R “pRRophic.” The response to erlotinib in the HR individuals was higher than the LR individuals (Figure 7(a)). In contrast, HR individuals displayed remarkably improved responses to 5-fluorouracil, cediranib, dasatinib, navitoclax, and sorafenib (Figures 7(b), 7(c), 7(d), 7(e), and 7(f)).

### 3.8. Single-Cell RNA-Seq Data Analysis

**Figure 8:**
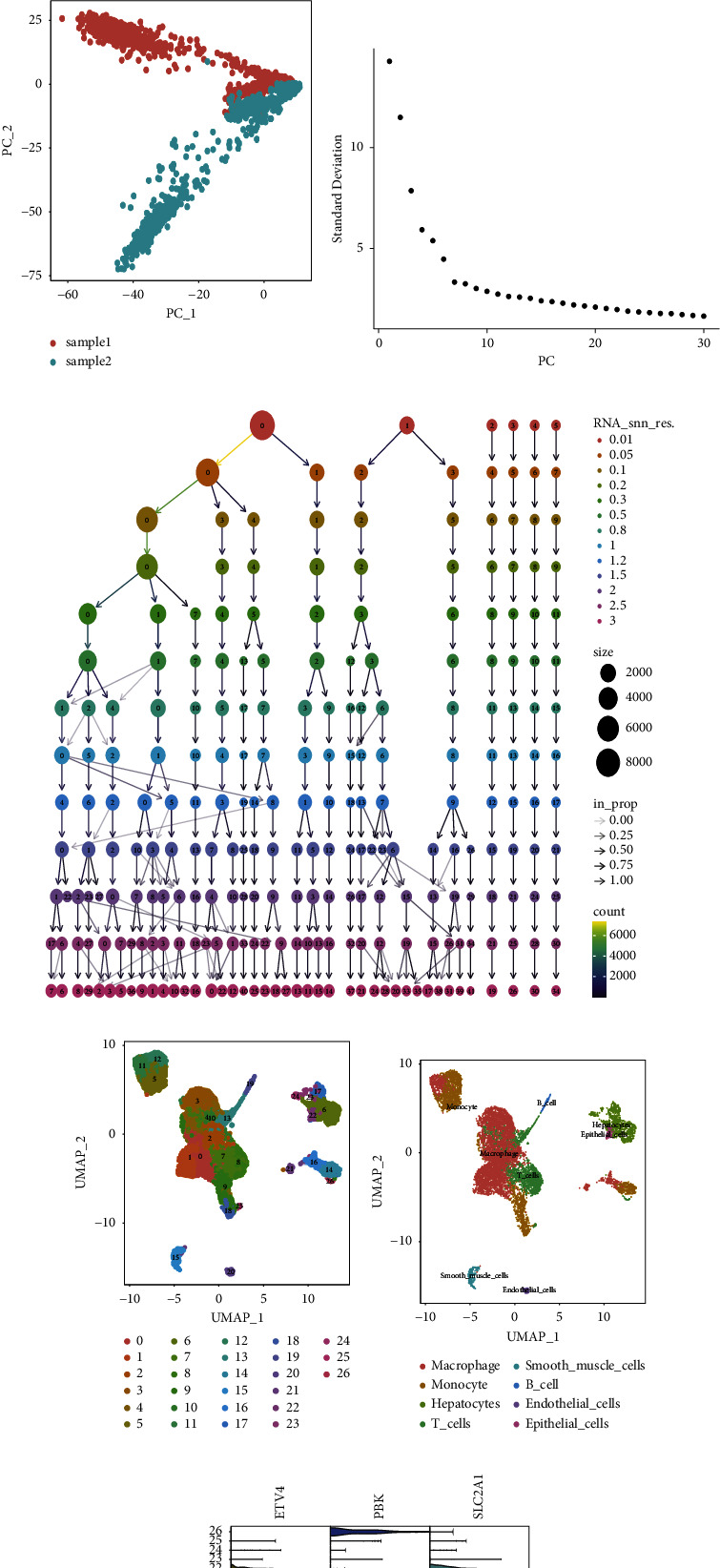
Single-cell RNA sequencing data analysis of risk genes for constructing gene signatures. (a) Quality control of single-cell RNA sequencing data. (b) Correlation analysis between sequencing depth and mitochondrial genes. (c) Red represents 2000 highly variable genes and highlights the top 10 highly variable genes. (d) Principal component analysis (PCA). (e) The single-cell gene expression profile was used to determine the elbow plot of the optimal principal component. (f) The appropriate resolution was selected based on the clustering tree. (g) The uniform manifold approximation and projection (UMAP) algorithm was used to display 25 cell subpopulations. (h) Twenty-five cell subpopulations were annotated as 10 subpopulations. (i) The expression of risk genes in various cell subpopulations. (j) The correlation between risk genes and cell subpopulations.

The single-cell RNA-seq dataset GSE166635 was analyzed to examine ETV4, PBK, and SLC2A1 expression levels in the TME. For quality control, unqualified cells were filtered out (Figure 8(a)). Sequencing depth was positively correlated with gene quantity (coefficient = 0.89) (Figure 8(b)). After data normalization, the top 2000 highly variable genes were selected (Figure 8(c)). The PCA technique was utilized to reduce dimensions (Figure 8(d)). Based on the elbow plot, 12 optimal principal components were selected (Figure 8(e)). A resolution of 1.5 was selected based on the clustering tree results (Figure 8(f)). The UMAP algorithm displayed the abundance of 27 cell subpopulations (Figure 8(g)). The Cellmaker database was used to query relevant genes, annotating 27 different cell subpopulations (Figure 8(h)). ETV4, PBK, and SLC2A1 expression levels were analyzed in various cell subpopulations (Figure 8(i)). SLC2A1 was significantly upregulated in the macrophage subpopulation (Figure 8(j)).

### 3.9. Validation of Risk Genes

**Figure 9:**
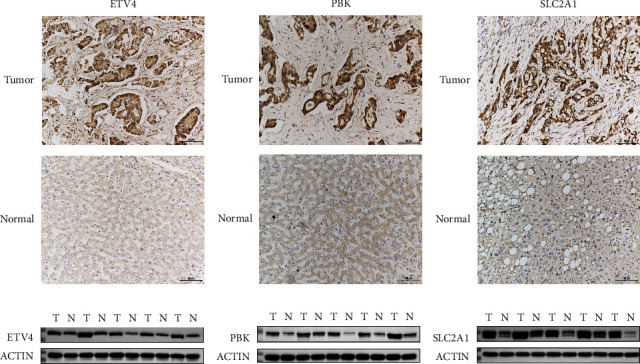
Validation of the protein expression levels of risk genes. (a–c) Immunohistochemical analysis of the ETV4, PBK, and SLC2A1 levels in tumor and noncancerous samples. (d–f) Western blotting analysis of ETV4, PBK, and SLC2A1 expression levels. A, D: ETV4; B,E: PBK; C,F: SLC2A1.

The protein expression levels of risk genes were validated using IHC analyses and western blotting. IHC analysis demonstrated that the ETV4, PBK, and SLC2A1 levels in HCC samples were upregulated when compared with those in paracancerous samples (Figures 9(a), 9(b), and 9(c)). Consistently, Western blotting revealed that the ETV4, PBK, and SLC2A1 levels were upregulated in HCC samples (Figures 9(d), 9(e), and 9(f)).

## 4. Discussion

This research delved into the mechanism of genes related to anoikis in HCC and established a clinical predictive model. Utilizing the prognostic genes linked to anoikis, the HCC study cohort was stratified into four subtypes (clusters A, B, C, and D). Cluster A was associated with the worst survival outcome. Subsequently, a clinical prognostic model was developed, comprising three genes (ETV4, PBK, and SLC2A1) selected from the pool of 23 prognostic genes related to anoikis, utilizing the LASSO regression model. The model accurately risk stratified patients with HCC. LR individuals displayed elevated survival rates relative to HR individuals. Strong positive and negative correlations were observed between the risk score and the percentages of activated and resting memory CD4+ T cells, respectively. LR individuals were predicted to benefit from erlotinib therapy. Meanwhile, HR individuals were predicted to benefit from 5-fluorouracil, cediranib, dasatinib, navitoclax, and sorafenib therapies. Hence, the clinical predictive model developed in this study demonstrates robust predictive capabilities, providing valuable guidance for clinical decision-making. Moreover, this clinical prognostic model accurately stratifies patients according to risk, facilitating accurate disease monitoring and treatment guidance. It aids in the selection of appropriate and targeted interventions, potentially improving treatment outcomes for patients with HCC.

GSVA demonstrated that cluster A exhibited a remarkable association with various pathways, encompassing the PPAR signaling and peroxisome pathways. Cluster B was significantly associated with different pathways, including homologous recombination and cell cycle pathways. Cluster C exhibited a remarkable association with metabolic pathways, including tyrosine, histidine, and tryptophan metabolism. The PPAR signaling pathway has been reported to be critically involved in multiple cellular processes, encompassing cell differentiation, inflammatory responses, modulation of glucose and lipid metabolism, immune modulation, and the intricate processes of tumorigenesis [27, 28]. PPAR comprises three main isoforms (PPAR*α*, PPAR*β*, and PPAR*γ*). Prior research has revealed that the dysregulation of the PPAR signaling pathway promotes the onset of different cancers, encompassing renal clear cell carcinoma, bladder cancer, and HCC [29–33]. Shuzhen Chen reported that 4-phenylbutyric acid upregulates PPAR-*α* through the activation of *β*-catenin signaling to initiate HCC stem cell formation [34]. Huayuan Liu et al. demonstrated that FNDC5 induces the transformation of tumor-associated macrophages from M1-type to M2-type. This transition is achieved by suppressing the initiation of NLRP3 inflammatory vesicles and facilitating the PPAR*γ* pathway, ultimately promoting the development of HCC [35]. This observation may elucidate the rationale behind the unfavorable survival outcomes observed in cluster A.

A clinical prognostic model containing three genes (ETV4, PBK, and SLC2A1) was developed. The clinical prognosis-predictive capability of the model was validated using ROC analysis. ETV4, PBK, and SLC2A1 expression levels in HR individuals were elevated relative to LR individuals. Consistently, IHC analysis highlighted that the expression of ETV4, PBK, and SLC2A1 in HCC samples was elevated relative to those in paracancerous samples. ETS transcription factors, which are members of an evolutionarily conserved family of transcription factors, have conserved ETS DNA-binding domains. Prior research has revealed that ETS transcription factors are involved in physiological activities, including cell differentiation, proliferation, development, and apoptosis [36, 37]. ETV4, a member of a subfamily of ETS transcription factors, is upregulated in HCC. In addition, ETV4 expression exhibits a negative association with the survival outcomes of individuals with HCC [38]. Previous research has demonstrated that ETV4 can facilitate the development of HCC by activating several key oncogenes and related signaling pathways, including the MMP1, uPAR, and Wnt/*β*-cyclin pathways [38–41]. Su et al. reported that ETV4 can enhance the angiogenic capacity of endothelial cells in the HCC microenvironment by modulating the transcriptional level of *MMP14*, promoting the development of HCC [42]. PBK, which functions as a mitotic kinase, regulates cell survival, proliferation, growth, apoptosis, and inflammation [43–45]. PBK upregulation exhibits diagnostic and prognostic relevance in cancers. PBK upregulation is linked to the development of ovarian plasma membrane and unfavorable prognosis in patients with ovarian plasma cystic adenocarcinoma, esophageal cancer, and gastric adenocarcinoma [46–48]. Previous studies have reported that PBK knockdown or treatment with PBK inhibitors suppresses tumorigenicity and exerts growth-inhibitory effects [49, 50]. Yang et al. demonstrated that PBK promotes HCC cell invasion and migration by the ETV4-uPAR signaling pathway [39]. The expression of SLC2A1 is upregulated in human tumors, such as colon and gastric cancers [51, 52]. SLC2A1 upregulation was significantly associated with poor prognosis in patients with HCC [53]. Peng et al. demonstrated that YAP1 inhibition decreased the SLC2A1-mediated Warburg effect, suppressing the development of HCC [54].

Somatic mutation analysis highlighted that the mutation frequency of TP53 in HR individuals was elevated relative to LR individuals. Wild-type TP53 protein initiates and promotes apoptosis in cells with DNA damage to prevent the aberrant proliferation of damaged cells [55]. However, *TP53* mutations inhibit apoptosis mechanisms, transforming damaged cells into cancer cells owing to apoptosis evasion. Mutant TP53 proteins lose their wild-type function and are unable to mediate apoptosis and cell cycle regulatory processes. Additionally, the nucleus may witness an accumulation of mutant TP53 proteins, a characteristic often regarded as a highly specific marker of malignancy [56]. The most common mutations in HCC are TP53 mutations [57]. TP53 assumes a crucial role in preserving genomic stability. A deficiency in the functional aspects of TP53 can result in centromere expansion, proliferation of aneuploid cells, and chromosomal instability (CIN) [34]. When TP53 mutations coexist with functional defects in the oncogene pRb or defects in the spindle checkpoint, it may contribute to the upregulation of CIN and genomic instability [58]. Previous studies have demonstrated that mutant TP53 proteins lose their tumor suppressor functions while acquiring functions that promote tumorigenesis [59]. These mutations may lead to enhanced tumor cell proliferation, invasion, metastasis, and treatment resistance. This suggests that the outcomes of the current research are consistent with those of earlier research, demonstrating the prognostic relevance of TP53 mutations in HCC. Therefore, the detection and evaluation of *TP53* mutations are important for treatment decisions and prognostic assessment in HR patients.

Several limitations are associated with this study. The analysis relied on data from public databases, and the available datasets provided limited information on the clinicopathological characteristics of patients. Consequently, it is essential to incorporate practical and critical factors for accurate predictions of survival outcomes in patients with HCC. Furthermore, the validation of risk gene expression levels was conducted through only IHC and western blotting analyses. To enhance the robustness of the findings, increasing the sample size is imperative, and the clinical value of this prognostic model should be verified through prospective studies.

## 5. Conclusions

In this research, four distinct anoikis-associated subgroups were identified in HCC, and a clinical prognostic model comprising three risk genes was established. This model demonstrated a high degree of accuracy in stratifying HCC patients, exhibiting excellent performance in predicting their survival outcomes. The insights gained from this research contribute valuable knowledge to the understanding of anoikis mechanisms in HCC, offering potential guidance for the development of personalized treatments for individuals with this disease.

## Data Availability

The datasets (TCGA-HCC; GSE76427) analyzed in this study were obtained from the Cancer Genome Atlas (TCGA, https://portal.gdc.cancer.gov) database and the Gene Expression Omnibus (GEO, https://www.ncbi.nlm.nih.gov/geo/).
